# Massive parallel sequencing as a new diagnostic approach for phenylketonuria and tetrahydrobiopterin-deficiency in Thailand

**DOI:** 10.1186/s12881-017-0464-x

**Published:** 2017-09-16

**Authors:** Pongsathorn Chaiyasap, Chupong Ittiwut, Chalurmpon Srichomthong, Apiruk Sangsin, Kanya Suphapeetiporn, Vorasuk Shotelersuk

**Affiliations:** 1 Center of Excellence for Medical Genetics, Department of Pediatrics, Faculty of Medicine, Chulalongkorn University, Bangkok, 10330 Thailand; 2 Excellence Center for Medical Genetics, King Chulalongkorn Memorial Hospital, the Thai Red Cross Society, Bangkok, 10330 Thailand; 3 Department of Orthopedics, Faculty of Medicine, Chiang Mai University, Chiang Mai, 50200 Thailand; 4 Division of Medical Genetics and Metabolism, Department of Pediatrics, Sor Kor Building 11th floor, King Chulalongkorn Memorial Hospital, Bangkok, 10330 Thailand

**Keywords:** Next generation sequencing, Exome, Hyperphenylalaninemia, Phenylketonuria, Tetrahydrobiopterin deficiency, Newborn screening

## Abstract

**Background:**

Hyperphenylalaninemia (HPA) can be classified into phenylketonuria (PKU) which is caused by mutations in the phenylalanine hydroxylase (*PAH*) gene, and BH4 deficiency caused by alterations in genes involved in tetrahydrobiopterin (BH4) biosynthesis pathway*.* Dietary restriction of phenylalanine is considered to be the main treatment of PKU to prevent irreversible intellectual disability. However, the same dietary intervention in BH4 deficiency patients is not as effective, as BH4 is also a cofactor in many neurotransmitter syntheses.

**Method:**

We utilized next generation sequencing (NGS) technique to investigate four unrelated Thai patients with hyperphenylalaninemia.

**Result:**

We successfully identified all eight mutant alleles in PKU or BH4-deficiency associated genes including three novel mutations, one in *PAH* and two in *PTS*, thus giving a definite diagnosis to these patients. Appropriate management can then be provided.

**Conclusion:**

This study identified three novel mutations in either the *PAH* or *PTS* gene and supported the use of NGS as an alternative molecular genetic approach for definite diagnosis of hyperphenylalaninemia, thus leading to proper management of these patients in Thailand.

## Background

Phenylketonuria (PKU) is an autosomal recessive metabolic disorder, characterized by progressive intellectual disability, mousy odor, autism, seizures, eczema and motor deficits [1]. The incidence of PKU in Caucasians is approximately 1:10,000 births [2]. Early diagnosis of the disease is beneficial since intellectual disability can be prevented by certain therapeutic intervention [3]. Generally, measuring blood phenylalanine levels provides a reliable and practical diagnosis for PKU. If the blood levels of phenylalanine exceed 120 μmol/l (2 mg/dl), the individual is considered to be hyperphenylalaninemia (HPA) and needs further diagnosis [4]. The majority of Caucasian patients with excessive phenylalanine levels are classified as classic PKU (MIM#261600), which is caused by loss-of-function mutations in the phenylalanine-4-hydroxylase (*PAH*) gene. About 2% of Caucasian cases are BH4-deficient [5], caused by mutations in the genes involved in BH4 biosynthesis consisting of 6-pyruvoyl-tetrahydropterin synthase (*PTS*), GTP cyclohydrolase I (*GCH1*), quinoiddihydropteridine reductase (*QDPR*), pterin-4-alpha-carbinolamine dehydratase 1 *(PCBD1*), and sepiapterin reductase (*SPR*) [5–7]. Interestingly, the incidence of PKU in Thailand is much less with a report of 1:212,535 in newborns [8] and the proportion of Thai patients with HPA who have BH4 deficiency remains unknown.

Although both PKU and BH4-deficiency patients show high blood phenylalanine levels and progressive intellectual disability, they respond to the treatment differently. Patients with PKU need additional supplement and are required to follow a diet that limits food with high phenylalanine such as dairy products, eggs, meat and fish [9]. This diet therapy is highly recommended to continue throughout their life to prevent behavioral disorders, cognitive, and emotional dysfunction [10]. On the other hand, patients with BH4 deficiency require BH4 supplement, generally 2–20 mg/kg/day [11, 12], as BH4 is also a cofactor in neurotransmitter synthesis, such as dopamine, serotonin, norepinephrine and epinephrine. Thus, deficiency of BH4 leads to not only elevated phenylalanine levels, but also deficiencies of these neurotransmitters [11]. Therefore, it is important for physicians to give an early and precise diagnosis, whether HPA patients are PKU or BH4-deficiency, to provide a proper treatment to the patients.

Neonatal screening for PKU in Thailand was started in 1996 by Department of Medical Sciences, Ministry of Public Health, aiming to early detect and provide treatment to PKU patients through a screening program [8]. The newborns’ blood samples were collected by heel prick on filter paper which was subsequently tested using the Guthrie method and/or the fluorometric method. The positive cases underwent plasma amino acid analysis using high performance liquid chromatography to determine phenylalanine levels [13]. Determination of urinary pterin levels can be used to screen BH4-deficiency. Unfortunately, it is not an integral part of newborn screening in Thailand, which may lead to late diagnosis in some cases [13].

Conventional genetic diagnosis for PKU and BH4 deficiency relies on Sanger sequencing of the entire coding regions of the responsible genes. A total of 42 exons of PKU, BH4-deficiency associated genes are needed to be analyzed. This approach is time consuming and labor intensive [14]. In contrast to Sanger sequencing, next-generation sequencing (NGS) can provide high throughput information from massive parallel sequencing from a single procedure [15] which is faster and more efficient. This technology has been widely used for research purpose but increasingly applied for genetic diagnosis, including PKU and BH4-deficiency [14, 16–19].

In this study, we apply next generation sequencing technique to investigate four unrelated Thai patients with hyperphenylalaninemia. The disease-causing variants were identified in all patients.

## Methods

### Patients

Patients I, II, III and IV are children from four unrelated non-consanguineous families. Their neonatal phenylalanine screening levels were 21.4, 53.11, 37.9, and 42.6 mg/dl, respectively (normal <20 mg/dl). After the diagnosis of hyperphenylalaninemia, all patients were treated with low-phenylalanine diet. For those diagnosed with BH4 deficiency, BH4 supplement was given. More clinical details are in Table 1.

**Table 1:** Clinical and molecular findings

Patient	I	II		III		IV	
Sex	F	M		F		F	
Age at definite diagnosis (mo)	1	21		6		1	
Age at last visit (mo)	51	36		16		5	
Development	normal	delayed		normal		normal	
Mutated gene	*PAH*	*PTS*	*PTS*	*PTS*	*PTS*	*PAH*	*PAH*
Genomic position (hg19)	12:103,248,934	11:112,099,388	11:112,101,362	11:112,103,916	11:112,104,166	12:103,234,294	12:103,249,009
dbSNP ID	novel	rs104894275	rs370340361	novel	novel	known [18]	rs62514927 [30]
Zygosity	homozygous	compound het	compound het	compound het	compound het	compound het	compound het
Reference allele (REF)	–	A	C	A	A	C	T
Alternate allele (ALT)	T	G	T	C	G	G	C
No. reads with REF in proband	0	25	55	21	14	114	55
No. reads with ALT in proband	51	31	46	16	5	101	33
Mutation type	frameshift ins	missense	missense	missense	missense	splice acceptor	exonic splicing enhancer
cDNA change	c.686_687insA	c.155A > G	c.200C > T	c.274A > C	c.326A > G	c.1200-1G > C	c.611A > G
Protein change	p.Asp229Glufs*54	p.Asn52Ser	p.Thr67Met	p.Asn92His	p.Asn109Ser	N/A	p.Tyr204Cys
Prediction SIFT	N/A	damaging	damaging	damaging	damaging	N/A	damaging
Prediction PROVEAN	N/A	deleterious	deleterious	deleterious	deleterious	N/A	neutral

Written informed consent for genetic analysis was obtained from the parents of the patients.

### Exome sequencing

Three milliliters of peripheral blood were taken from each patient after informed consent. Genomic DNA was extracted by using Gentra Puregene Blood Kit, Qiagen (Qiagen, Hilden, Germany). The extraction process was done according to the manufacturer’s protocol. Genomic DNA from four patients were sent for exome sequencing using service from Macrogen, Inc. (Seuol, South Korea). The samples were prepared according to Agilent SureSelect Target Enrichment Kit (Agilent Technologies, Santa Clara, CA) preparation guide. The captured libraries were sequenced with Illumina HiSeq 2000 or HiSeq2500 Sequencer. The result sequences were aligned to the human genome reference sequence (UCSC hg19) using Burrows-Wheeler Alignment (bwa-0.7.10, http://bio-bwa.sourceforge.net/) [20]. Picard software (picard-tools-1.118, http://broadinstitute.github.io/picard/) was used for marking and removing duplicated sequences. Genome Analysis Toolkit (GATK3.v4, https://www.broadinstitute.org/gatk/) [21] was used for data quality assessment, genotyping and variant calling. Finally, SnpEff_v.4.1 (http://snpeff.sourceforge.net/) [22] was used for variant annotation.

### Genotype analysis

All homozygous and compound heterozygous variants with <1% allele frequency in global population and located on genes associated with hyperphenylalaninemia (*PAH, PTS, GCH1, QDPR, PCBD1, SPR* and *GCHFR*) were included. The variants with >1% of the 165 unrelated Thai exomes, our in-house database, were excluded. The novel variants were confirmed by Sanger sequencing. The patients’ genomic DNA was amplified by polymerase chain reaction (PCR), using primers specific to the site of the mutation (Table 2). The PCR products were then sent for Sanger sequencing by Macrogen Inc. (Seoul, South Korea). The sequences were analyzed by Gene Codes Sequencer software (v.5.4.1) (Gene Codes Corporation, MI). The sequencing data were compared to unaffected control sequences. ClustalX 2.1 (http://www.clustal.org/clustal2/) [23] was used for amino acid conservative analysis. ExAC Browser: Exome Aggregation Consortium Database (http://exac.broadinstitute.org/) was used to confirm the novelty of the variants [24].

**Table 2:** Primer sequences for Sanger sequencing of novel mutations

Primer name	Sequence	Tm (°C)
*PAH*_E6_F	5′-GAT GGC AGC TCA CAG GTT CT-3′	60.5
*PAH*_E6_R	5′-CTT GTC TTC CCC TTC CCT CT-3′	60.5
*PTS*_E5-6_F	5′-TGA TAA GGT GAG GTT TAG AGG C-3′	60.1
*PTS*_E5-6_R	5′-CTC CAG AGC ACA ATG TGT ACG-3’	61.2

## Results

**Fig. 1:**
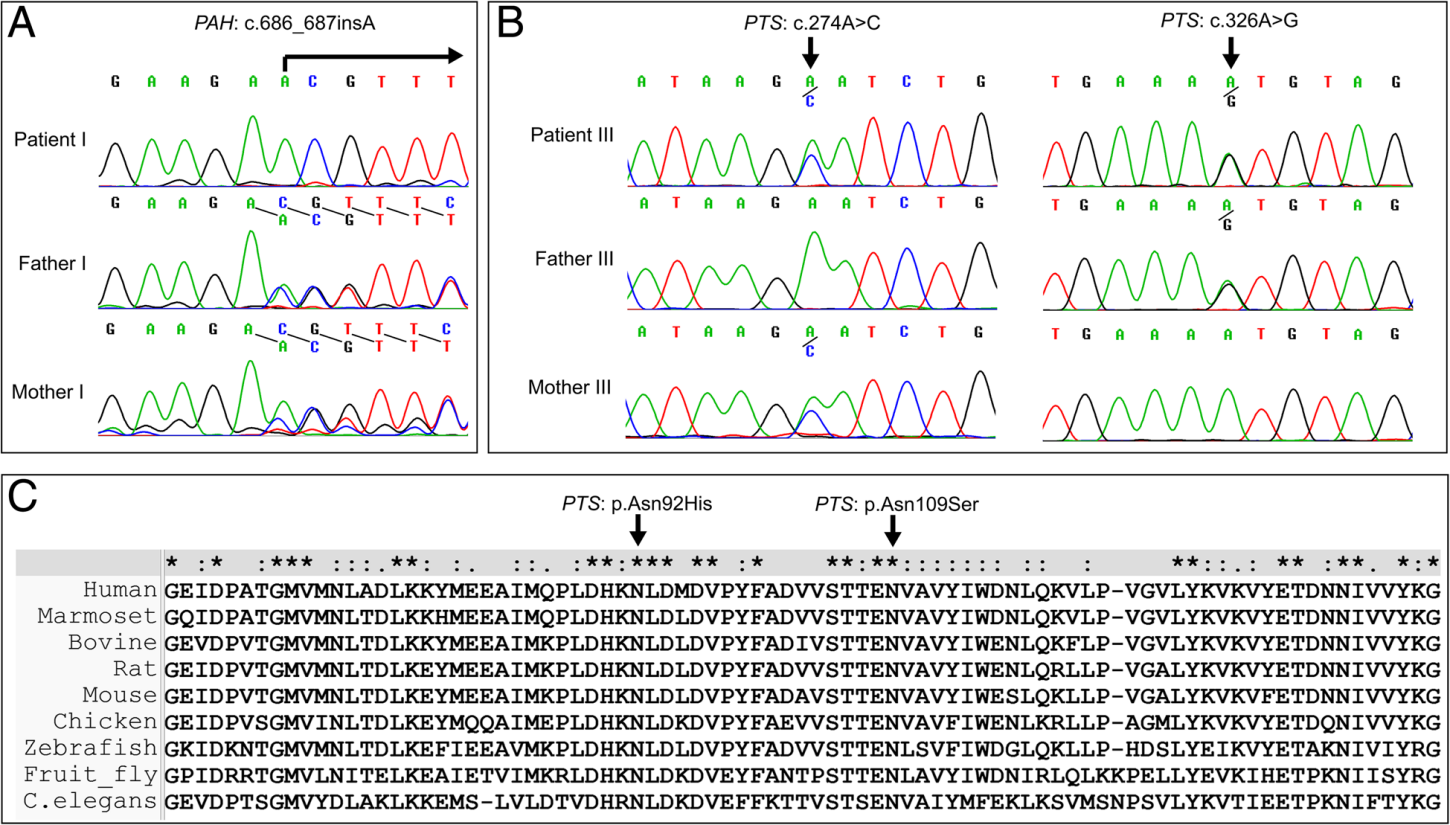
Chromatogram of novel mutations in Patient I (**a**) and Patient III (**b**) and amino acid conservation for novel missense mutations in Patient III (**c**)

All four patients showed approximately 20,000 DNA variants across the exome (Table 3). Mutation analysis successfully identified all eight mutant alleles in PKU or BH4-deficiency associated genes. Patients I and IV had mutations in the *PAH* gene, while Patients II and III had mutations in the *PTS* gene. The detailed characteristics of the mutations are summarized in Table 1. Sanger sequencing of the novel mutations confirmed the presence of the mutations found by exome sequencing (Fig. 1a and b). The amino acid alignment from ClustalX revealed that the two novel missense mutations in the *PTS* gene identified in Patient III were highly conserved (Fig. 1c).

**Table 3:** Summary of exome sequencing data of the patients from each family

	Patient I	Patient II	Patient III	Patient IV
Mean coverage depth of target regions (X)	60×	64×	47×	76×
% of captured regions with coverage >10	97%	96%	96%	94%
Total number of coding SNPs	19,887	19,543	19,599	22,514
Total number of coding INDELs	472	472	469	619
Number of homozygous variants^a^	1	0	0	0
Number of compound heterozygous variants^a^	0	2	2	2

^a^Only variants with population allele frequency < 1%, and located in *PAH, PTS, GCH1, QDPR, PCBD1, SRP* and *GCHFR* were counted. Variants that are presented in >1% of the in-house Thai exome database were not included

## Discussion

Newborn screening program for HPA is important since severe intellectual disability from PKU or BH4 deficiency is preventable with proper treatment. Although Thailand has established Neonatal Screening Program for PKU for many years, screening positive patients do not undergo a test for BH4-deficiency due to the unavailability of pterin analysis in Thailand [25]. In addition, BH4 loading test cannot distinguish patients with BH4 deficiency and BH4-responsive PKU [5]. The conventional Sanger sequencing has been the gold standard test for a molecular approach in the genetic diagnosis of inherited disorders. This direct approach is suitable for sequencing hot-spot point mutations or small genes [26]. However, it is rather costly and time consuming for diagnosis of genetic disorders which involve large genes or multiple genes, including genes associated with PKU and BH4-deficiency. The costs associated with NGS are rapidly decreasing. Therefore, we utilize NGS as a diagnostic tool in our study as it has been demonstrated to be effective in several previous reports [14, 16–19].

All eight mutant alleles were successfully identified. Of these, three have never been previously reported. Patient I has a novel homozygous frameshift mutation (c.686_687insA) in the *PAH* gene. Patient II has two compound heterozygous missense mutations (c.155A > G and c.200C > T) in the *PTS* gene. The c.155A > G mutation has been previously reported in Chinese patients [27] and the c.200C > T in Italian patients [28]. Patient III has two novel compound heterozygous missense mutations (c.274A > C and c.326A > G) in the *PTS* gene. Both mutations are predicted to be damaging and deleterious from prediction softwares, SIFT and PROVEAN. Patient IV has compound heterozygous, one exonic splice site enhancer (c.611A > G) and one splice acceptor (c.1200-1G > C) mutations in the *PAH* gene. The c.611A > G mutation has been reported in Asian [29] and European patients [30] and the c.1200-1G > C has been recently reported in Chinese patients [18]. Therefore, Patients I and IV were diagnosed as classic PKU and were continued on low phenylalanine diet, while Patients II and III were diagnosed with BH4-deficiency and were given BH4 supplement.

In order to reduce the number of identified variants from NGS data, investigators often need to apply filtering criteria to exclude low quality variants [31]. This parameter could be depth of coverage. Interestingly, one of the variants in this study at position chr11:112,104,166, c.326A > G in the *PTS* gene has total read depth of just 19 reads, with an alternate read of only 5 reads. This can be considered as very low read depth in other studies, and may have been removed from analysis [32, 33]. We would not be able to find causative mutations in patient III if we discard this variant based on the variant quality alone. In addition, despite no consanguinity history in all four families, Patient I has a frameshift homozygous mutation in the *PAH* gene. From additional interview, it appeared that both of her parents came from the same district of the same province. It might not be uncommon for patients with non-consanguineous parents to have homozygous mutations.

Patients II and III received BH4 therapy after the definite diagnosis was made. Patient II however had delayed development and recurrent seizure treated with anticonvulsive drugs, phenytoin and levetiracetam. At his last visit when he was three years old, he still had delayed development and occasional abnormal movement (orofacial dyskinesia and chorea). This might be due to the age of Patient II when diagnosed, which was one year and nine months old. On the other hand, Patient III was given BH4 supplement at the age of 6 months after the diagnosis was made. The patient showed normal development at her last visit when she was one year and four months old. This demonstrated the importance of providing early and precise diagnosis to HPA patients.

Only 2% of Caucasian patients with HPA are BH4 deficient. Interestingly, of our four consecutive patients, two are BH4 deficient. The total number of patients are very small. However, since the treatment to prevent intellectual deficit is different between both types, it is warrant to determine the proportion of PAH and BH4 deficiency among Thai patients with HPA. If a significant percentage of Thai patients with HPA have BH4 deficiency, it should be justified to perform genetic testing using NGS as soon as HPA is identified.

## Conclusion

This report represents the first study in Thailand to successfully use next-generation sequencing to detect causative mutations in PKU and BH4-deficiency cases, which allow physicians to provide a precise diagnosis and proper effective treatment to the patients. This approach is ready to substitute conventional sequencing for genetic diagnosis of patients with hyperphenylalaninemia.

## Abbreviations

BH4: tetrahydrobiopterin
HPA: hyperphenylalaninemia
NGS: next generation sequencing
PAH: phenylalanine hydroxylase
PKU: phenylketonuria
